# Is high aerobic workload at work associated with leisure time physical activity and sedentary behaviour among blue-collar workers? A compositional data analysis based on accelerometer data

**DOI:** 10.1371/journal.pone.0217024

**Published:** 2019-06-06

**Authors:** Charlotte Lund Rasmussen, Javier Palarea-Albaladejo, Mette Korshøj, Nidhi Gupta, Kirsten Nabe-Nielsen, Andreas Holtermann, Marie Birk Jørgensen

**Affiliations:** 1 National Research Centre for the Working Environment, Copenhagen, Denmark; 2 Section of Social Medicine, Department of Public Health, University of Copenhagen, Copenhagen, Denmark; 3 Biomathematics and Statistics Scotland, Edinburgh, United Kingdom; 4 Department of Sports Science and Clinical Biomechanics, University of Southern Denmark, Odense, Denmark; 5 Department of Forensic Science, University of Copenhagen, Copenhagen, Denmark; Teesside University/Qatar Metabolic Institute, UNITED KINGDOM

## Abstract

**Objective:**

This study aimed to investigate the hypothesized negative association between duration of work time spent at a high relative aerobic workload and leisure time movement behaviours among blue-collar workers.

**Methods:**

This was a cross-sectional study based on heart rate and accelerometer data from 803 blue-collar workers (447 men and 356 women). Relative aerobic workload was measured as percentage of heart rate reserve during work (%HRR). Leisure time movement behaviours were expressed in terms of leisure time spent in sedentary and active behaviours in uninterrupted bouts (i.e. <10 min, ≥10–30 min and >30 min). Compositional regression and isotemporal substitution models were used to assess the association between the predominance of work time spent at ≥40%HRR and leisure time spent in sedentary and active bouts. All analyses were stratified by sex.

**Results:**

For men, there was no statistically significant association between the predominance of work time spent at ≥40%HRR and leisure time movement behaviours. Among women, the predominance of ≥40%HRR at work was negatively associated with relative leisure time spent in ≥10 min bouts of active behaviour ($\hat{\beta }$ = -0.21, p = 0.02) and a theoretical 15 min reallocation of work time from <40%HRR to ≥40%HRR was estimated to decrease active behaviour by 6 min during leisure time.

**Conclusion:**

Our result highlights the need for considering work-related barriers for an active leisure time in high-risk populations. Longitudinal studies are warranted to disentangle the relationship between physically demanding work characteristics and leisure time movement behaviours in such populations.

## Introduction

Workers with high aerobic workloads have increased risk of cardiovascular disease and all-cause mortality [1–4]. The physiological mechanism is likely related to the characteristics of physically demanding job tasks which involve activities such as heavy lifting, pushing and pulling [2,5]. Performing such strenuous activities over prolonged time periods (e.g. 8 hours/5 days a week) imposes a high circulatory strain and subsequent risk of cardiovascular impairments [6–9]. Accordingly, for an 8-hour workday having a relative aerobic workload of 30–40% is considered a high aerobic workload [10,11]. High cardiorespiratory fitness could protect against these detrimental effects by reducing the relative aerobic workload when performing physically demanding job tasks [12–14].

Leisure time physical activity is typically performed in short bouts of high-intensity activities followed by adequate time for recovery and is found to enhance cardiorespiratory fitness [15]. In contrast, occupational physical activities are often performed without sufficient recovery, thereby not resulting in enhanced cardiorespiratory fitness [16,17]. In fact, hours of continuous physically demanding work each day is likely to cause fatigue [18]. Consequently, workers within manual jobs are likely to spent leisure time being sedentary instead of engaging in high-intensity activities[19,20]. Accordingly, we hypothesized high aerobic workload to be a barrier for performing bouts of health-enhancing physical activities and to increase the need for prolonged periods of sedentary leisure time. Given the accumulating evidence on health impairments associated with long, uninterrupted periods of sedentary behaviour and lack of bouted physical activities, such leisure time movement pattern could have severe health impact in an already high-risk population [21–25].

To our knowledge, no study has assessed the association between high aerobic workload and leisure time movement behaviours using device-based measurements and only two studies have investigated such association, both using self-reported measurements of leisure time physical activities [26,27]. However, self-reported measures of physical activities can be biased [28]. For example, individuals with higher cardiorespiratory fitness have been found to over-report physical activity levels more than those with lower cardiorespiratory fitness [29]. Accordingly, device-based measurements of physical activity have been recommended [28]. Moreover, time spent on physical activities at work and leisure time defines mutually exclusive and exhaustive parts of daily time awake. Consequently, these times are not independent of each other and, rather than analysing them in isolation, it is recommended to target all activities synergistically [30]. Proportions of daily time spent in each behaviour represent so-called compositional data for which dedicated statistical methodology has been developed [31,32]. Recently, this methodology has been successfully introduced in physical activity research [30,33,34]. Accordingly, the aim of our study was to investigate the association between device-based measured high aerobic workload at work and leisure time movement behaviours in a group of blue-collar workers, using compositional data analysis.

## Materials and methods

### Study design, study population and data collection

This study was based on cross-sectional data from the Danish Physical ACTivity cohort with Objective measurements (DPhacto) [35] and the New Method for Objective Measurements of Physical Activity in Daily Living (NOMAD) study [36]. The data collection and procedures in the two studies were identical, enabling merging of the two datasets.

The study population consisted of blue-collar workers from Danish companies within transportation, cleaning, manufacturing, construction, road maintenance, garbage disposal, assembly, mobile plant operator, and health services [35,36]. Eligible workers were employed for at least 20 hours/week; between 18–65 years old; had a blue-collar job; and given voluntary consent to participate. Workers were excluded if they were pregnant, had fever on the day of testing or allergy to adhesives.

Data were collected over four consecutive days and included a health check, questionnaire, and accelerometer and heart rate measurements over 24 hours a day [35,36]. Data collection and procedures have been described previously [35,36]. In short, eligible workers were invited to complete a questionnaire and to participate in a health check, consisting of anthropometric measurements and a physical health examination. Participants were asked to wear accelerometers and heart rate monitors for a minimum of two consecutive workdays and to complete a diary reporting time at work, time in bed and non-wear time. Daily work hours and leisure time were defined from the participants’ diary information.

**Fig 1:**
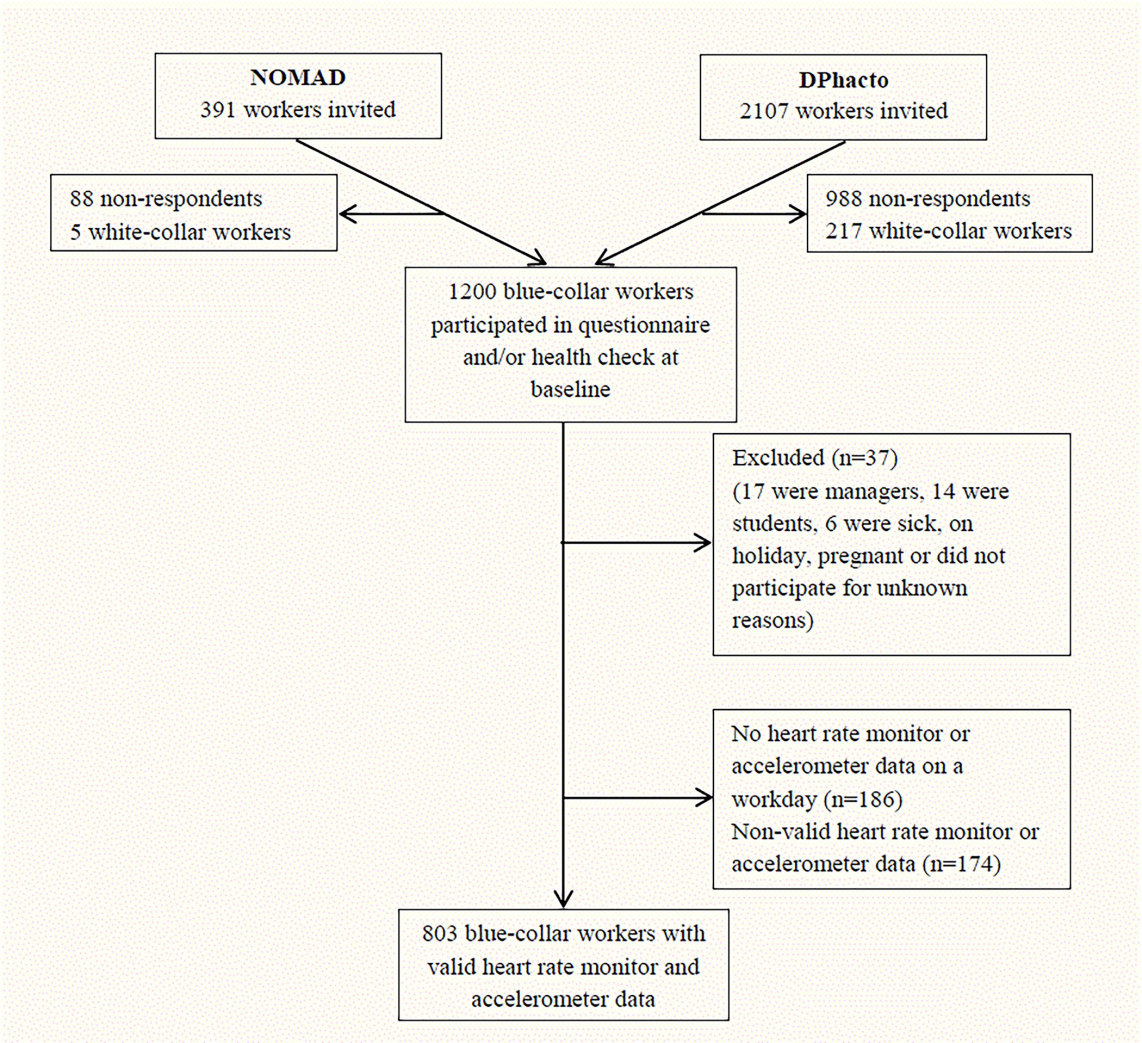
Flowchart of participants in the NOMAD and DPhacto study included in the current paper.

Only workers with at least one day of valid device-based measurements were included. A valid day consisted of having both a) heart rate measurement of ≥4 hours or 75% of the individual’s average work time and b) accelerometer measurement of ≥4 hours or 75% of the individual’s average leisure time awake. Time in bed at night was excluded from the analyses. Fig 1 shows the flow chart of the study population. A total of 1200 blue-collar workers participated in the baseline questionnaire and/or health check. Of these, 37 were excluded because they were managers, students, on holiday, pregnant or for unknown reasons; 186 did not have heart rate and/or accelerometer data; and 174 did not fulfil the criterion of having one valid day of device-based measurements. Thus, a total of 803 blue-collar workers (447 men and 356 women) were included in the analyses.

### Ethical considerations

The DPhacto and NOMAD studies were approved by the local Ethics Committee of the Capital region of Denmark (file number H-2-2012-011[35] and file number H-2-2011-047 [36], respectively). Both studies were conducted according to the Helsinki declaration and all data were anonymized in relation to individuals and workplaces.

### Measurements

#### Measurement of relative aerobic workload

Heart rate was measured using Actiheart (Camntech, Cambridge, United Kingdom), placed at the chest at one of the two standardized positions [37], consisting of two electrodes connected by a short lead attached to the skin by two standard electrocardiography pads (Ambu, Blue sensor VL-00-S/25) [38]. Data were downloaded in the Actiheart software (version 4.0.100) [39] and analysed by a custom-made MATLAB program Acti4 (The National Research Centre for the Working Environment, Denmark and The Federal Institute for Occupational Safety and Health, Germany (BAuA))[40].

Heart rate data were filtered and checked for errors according to an earlier described protocol [41]. In brief, inter-beat intervals corresponding to <36 or >200 beats/min were considered as physiological outliers and excluded [41]. Moreover, heart rate measurements including > 50% beat error were excluded. Relative aerobic workload was estimated based on the heart rate reserve (%HRR), which is a well-established estimate of the aerobic workload on the body depending on the work demand and the individual’s cardiorespiratory fitness [42]. Heart rate reserve was defined as the difference between estimated maximal heart rate (HR_max_) and sleeping heart rate (SHR) (HRR = HR_max_−SHR) for each worker [43]. HR_max_ was determined by the Tanaka equation [44] and SHR was defined as the minimum heart rate of an average of ten beats/min during time in bed at night [45]. The mean relative aerobic workload was then calculated as the percentage of estimated HRR (mean heart rate during work / HRR*100 = %HRR). Generally, for an 8-hour workday having a HRR of 30–40% is considered a high aerobic load [10,11]. Thus, we defined high aerobic workload as work hours spent with ≥40%HRR.

#### Accelerometer measurements of leisure time physical activity and sedentary behaviour

Leisure time movement behaviours were assessed using data from one tri-axial ActiGraph GT3X+ accelerometer (Actigraph, Florida, U.S.A). The accelerometer was placed on the right thigh using double-sided adhesive tape (3 M, Hair-Set, St. Paul, Minnesota, USA) and Fixomull (Fixomull BSN medical GmbH, Hamburg, Germany) [46]. Accelerometer data were downloaded using Actilife Software version 5.5 [47] and analysed using the Acti4 program. The Acti4 program enables identification of physical activity types and postures (i.e. cycling, stair climbing, running, walking, standing, sitting and lying) with high sensitivity and specificity using angles from the accelerometer axis and standard deviation of mean acceleration [40,48]. Sedentary behaviour during leisure time was defined as time spent sitting and/or lying. Leisure time spent active was defined as time spent standing, walking, stair climbing, running or cycling.

Temporal patterns of physical activities and sedentary behaviour during leisure time were measured and quantified using exposure variation analyses (EVA). EVA enabled identification of uninterrupted periods of different durations of specific activities and sedentary behaviour. The temporal patterns of sedentary behaviour were expressed as average leisure time spent in short (<10 min), moderate (≥10–30 min) and prolonged (>30 min) uninterrupted periods (minutes/day). Active behaviour was expressed as average leisure time spent in short (<10 min) or moderate (≥10 min) uninterrupted periods (minutes/day). The chosen temporal patterns comply with recommendations from the American Physical Activity Guidelines 2018 based on current evidence on sedentary and active behaviour bout lengths to avoid health impairments [21–25].

#### Covariates

Sex and age of the workers were determined from each worker’s unique Danish civil registration number (CPR-number). Body Mass Index (BMI) was calculated as body mass in (kg) divided by height (m) squared (kg/m^2^). Information about prescribed heart or lung medicine intake as obtained by the question: *“Do you take prescribed medication for heart or lung diseases*?*”*. Job seniority was determined by the question: *“For how long have you had the kind of occupation that you have now*?*”*. Information on shift work was assessed using the question: *“At which time of the day do you usually work in your main occupation*?*”* with 3 response categories; fixed day work; night/varying working hours with night; and other. The variable was dichotomised into workers with fixed day work and workers with no-fixed day work (including shift work and other).

### Statistical analyses

Compositional regression analysis was used to estimate the association between %HRR at work and leisure time movement behaviours. All analyses were performed in R version 1.1.3 [49], using the *compositions* [50], *robCompositions* [51] and *zCompositions* [52] packages.

Each worker’s average daily time use was conceptualized as consisting of two compositions i.e. work and leisure time. Daily work period was treated as a 2-part composition, consisting of time spent at <40%HRR and ≥40%HRR. Leisure time was treated as a 5-part composition, consisting of time spent on prolonged sedentary bouts (i.e. >30 min), moderate sedentary bouts (i.e. ≥10–30 min), short sedentary bouts (i.e. <10 min), short active bouts (i.e. <10 min) and moderate active bouts (i.e. ≥10 min). Six workers had zero leisure time spent in moderate active bouts and one worker had zero leisure time spent in moderate sedentary bouts. These zero observations were assumed to be due to limited sampling and treated as missing data. They were imputed by expected values using the log-ratio Expectation-Maximization (EM) algorithm based on the information in the covariance structure of the observed data set [53].

#### Compositional descriptive statistics

Compositional geometric means were calculated for the %HRR and leisure time compositions to describe the central tendency of the data [31,32]. They were obtained by computing the geometric mean of each individual part of the respective compositions and then normalising (closing) these vectors of geometric means to be expressed in units relative to the workers’ average daily work and leisure time (i.e. 442 min and 519 min, respectively). The pair-wise variation matrix and total variance of the leisure time composition were calculated as compositional summaries of data variability [31,32]. The pair-wise variation matrix indicates the co-dependence between the parts of the leisure time composition in terms of proportionality, with values close to 0 indicating that two parts are highly co-dependent. The total variance was decomposed into contributions from each part of the leisure time composition.

#### Isometric log-ratio (ilr) coordinates and compositional linear regression

A detailed description of the compositional multivariate linear regression and isotemporal substitution methods has been reported previously [54]. In short, the %HRR and leisure time compositions were represented using isometric log-ratio (*ilr*) coordinates. For the 2-part work composition, one ilr-coordinate expressed the relative importance (or predominance) of work time spent with ≥40%HRR relative to work time spent with <40%HRR. For the leisure time composition, we used so-called pivot ilr coordinates, by which all the relative information of the first part of the composition (with respect to the geometric mean of the remaining parts) is included in the first ilr-coordinate [55]. The parts were then sequentially rearranged to place each leisure time movement behaviour bout at the first position once and the corresponding ilr-coordinate sets were computed. In this way, the relative importance of each part was sequentially represented in the first ilr-coordinate of a set for subsequent statistical significance testing through regression analysis.

The strength and direction of the associations between the predominance of work time at ≥40%HRR and leisure time movement behaviour bouts were estimated using compositional multivariate linear regression models. In all models, the ilr-coordinate for the workers’ %HRR composition was given as the exposure variable and the set of ilr-coordinates of the workers’ leisure time composition defined the outcome variables. Five regression models were then fitted, each one isolating the relative importance of one of the leisure time movement behaviour bouts with respect to the others in the first ilr-coordinate (denoted by ilr_1_) as described above.

Studies have shown men and women to differ in work tasks and leisure time behaviours [56,57]. In line with this, we have previously found differences in work and leisure time physical activities within the same study population as in the current study [54]. Thus, all analyses were stratified by sex. Based on literature and theoretical considerations of potential confounders, models were adjusted for age [17,20], average work hours [58] and heart or lung medicine intake (reference group = none). Average total work hours was calculated using the logarithm of the geometric mean of time spent on each part of the %HRR composition multiplied by $\sqrt{D}=\surd 2$ [59]. Regression beta-coefficients and standard errors were estimated for the five regression models. For each model, 2-sided Wald test p-values were used to determine if the predominance of %HRR was statistically significantly associated with the predominance of the leisure time movement behaviour bout represented by the first ilr-coordinate, based a 5% significance threshold. The assumptions of normality and homoscedasticity of the residuals were assessed for all models by visual inspection of plots of residuals versus predicted values and quantile-quantile plots.

#### Compositional isotemporal substitution models

Compositional isotemporal substitution models were used to estimate the potential effect of reallocating work time spent with <40%HRR to ≥40%HRR, following the methods described in Dumuid et al. (2017) and Lund Rasmussen et al. (2018). This analysis was performed in three steps. Firstly, an expected leisure time composition was estimated based on the workers’ average %HRR composition. Secondly, a new %HRR composition was constructed by reallocating time spent with <40%HRR to ≥40%HRR from 15 min to 60 min in 15-min increases. Thirdly, expected changes in the leisure time composition were derived by taking the inverse ilr-transformation of the leisure time movement behaviour ilr-coordinates estimated by the reference baseline and new %HRR compositions and then calculating change in leisure time movement behaviours. Quantile-based bootstrap 95% confidence intervals [60,61] of the expected changes in the LTPA composition were estimated based on 1000 bootstrap resamples generated at random by sampling from the original dataset with replacement.

## Results

### Study population

Table 1 shows the baseline characteristics of the study population, stratified by sex. Among men, the average age was 43.9 (SD = 10.8) years; average BMI was 27.0 kg/m^2^ (SD = 4.3); 64% were smokers; 8% used prescribed heart or lung medicine; and the majority worked within manufacturing (69%). Among women, the average age was 46.8 (SD = 8.6) years; average BMI was 27.1 kg/m^2^ (SD = 5.4); 67% were smokers; 9% used prescribed heart or lung medicine; and most of the women worked in manufacturing (50%).

**Table 1:** Baseline characteristics of the study population, stratified by sex.

Variables	Men (n = 447)				Women (n = 356)			
	N	%	Mean (SD)	Range	N	%	Mean (SD)	Range
Age in years	447	100	43.9 (10.8)	[18.0;68.0]	356	100	46.8 (8.6)	[21.0;68.0]
Seniority in years	428	96	13.9 (10.9)	[0.0; 45.0]	336	94	13.0 (10.1)	[0.1; 48.0]
Overall health (1–5)^*A*^	437	98	2.2 (0.6)	[1.0; 5.0]	349	98	2.3 (0.7)	[1.0; 5.0]
BMI in kg/m^2^	439	98	27.0 (4.3)	[18.9;45.1]	352	99	27.1 (5.4)	[16.2;43.8]
Aerobic capacity (ml O2/min/kg)	353	79	33.7 (9.0)	[13.9;66.9]	251	71	29.9 (8.5)	[13.6; 68.9]
Alcohol consumption (units/week)	443	99	4.8 (6.2)	[0.0; 40.0]	350	98	1.8 (2.5)	[0.0; 18.0]
Days with valid heart rate monitor measurements	447	100	2.4 (0.9)	[1.0; 5.0]	356	100	2.4 (0.9)	[1.0; 5.0]
Days with valid accelerometer measurements	447	100	2.7 (1.0)	[1.0; 5.0]	356	100	2.5 (0.9)	[1.0; 5.0]
Cohort								
NOMAD	101	23			89	25		
DPhacto	346	77			267	75		
Fixed day job	341	76			279	78		
Skilled workers	227	51			119	33		
Smokers	288	64			237	67		
Prescribed heart or lung medicine intake	34	8			32	9		
Working sector								
Cleaning	18	4			126	35		
Manufacturing	311	69			178	50		
Transportation	55	12			2	1		
Health Service	0	0			16	5		
Assemblers	2	1			28	7		
Construction	26	6			0	0		
Garbage Collectors	16	4			0	0		
Mobile Plant Operators	6	1			0	0		
Other^B^	13	3			9	2		

BMI = body mass index. SD = standard deviation.

^A^High scores indicate higher self-reported heath.

^B^Includes general office clerks and other elementary workers.

### Compositional descriptive statistics

Compositional geometric means (CGMs) of the %HRR and leisure time compositions stratified by sex are presented in Table 2. Both men and women spent the majority of their work time at <40%HRR (90% and 89% time, respectively). The average distribution of leisure time spent in sedentary and active bouts were similar for men and women.

**Table 2:** Compositional geometric mean (CGM) for percentage heart rate reserve at work (%HRR) and leisure time movement behaviour bouts (in minutes/day and %), stratified by sex.

	Men (n = 447)		Women (n = 356)	
**%HRR at work (CGM)**				
	*Min*.*/day*	*%*	*Min*.*/day*	*%*
<40%HRR	400	90	396	89
≥40%HRR	42	10	46	11
**Leisure time bouts (CGM)**				
	*Min*.*/day*	*%*	*Min*.*/day*	*%*
SB ≥30 min	162	31	143	27
SB ≥10–30 min	115	22	102	20
SB<10 min	66	13	66	13
Active<10 min	84	18	96	19
Active≥10 min	82	16	111	22

Active = standing, walking, running, stair climbing, and cycling. CGM = compositional geometric mean. %HRR = percentage heart rate reserve. SB = sedentary behaviour (sitting and lying). Time-use of %HRR was closed to the workers’ average daily work hours (442 minutes). Time-use of leisure time composition was closed to workers’ average daily leisure time (519 minutes).

Table 3 displays the variation matrix of the leisure time composition for men and women. For both sexes, the strongest association was observed between short sedentary bouts and short active bouts during leisure time (log-ratio variances τ = 0.11 and τ = 0.11, respectively). Moreover, for both men and women the moderate active bouts category was the main contributor to the total variation (56% and 42%, respectively), indicating that leisure time spent on moderate active bouts varied considerably.

**Table 3:** Compositional variation matrix for leisure time spent on movement behaviour bouts, stratified by sex.

	Men (n = 447)						Women (n = 356)					
	SB >30 min	SB ≥10–30 min	SB <10 min	Active <10 min	Active ≥10 min	Var-clr (%)	SB ≥30 min	SB ≥10–30 min	SB <10 min	Active <10 min	Active ≥10 min	Var-clr (%)
SB >30 min	0.00					0.47 (23)	0.00					0.49 (24)
SB ≥10–30 min	0.68	0.00				0.15 (8)	1.12	0.00				0.42 (21)
SB <10 min	0.81	0.24	0.00			0.15 (8)	0.84	0.62	0.00			0.15 (8)
Active <10 min	0.67	0.21	0.11	0.00		0.10 (5)	0.73	0.53	0.11	0.00		0.10 (5)
Active ≥10 min	2.23	1.73	1.64	1.57	0.00	1.14 (56)	1.77	1.79	1.23	1.17	0.00	0.85 (42)
Total var						2.01 (100)						2.01 (100)

Active = standing, walking, running, stair climbing, and cycling. SB = sedentary behaviour (sitting and lying). Total var = total variance of the composition. Var-clr (%) = absolute and percentage (%) contribution of each part to the total variance. Values close to 0 indicate that two parts are nearly proportional (highly co-dependent) and thus, their log-ratio is nearly constant.

### Compositional multivariate linear regression analyses

For men, we found no statistically significant association between the predominance of work time spent with ≥40%HRR and of any type of leisure time movement behaviour bouts (Table 4; p > 0.05 in all cases). For women, relative work time spent with ≥40%HRR was positively associated with the predominance of short sedentary bouts ($\hat{\beta }$ = 0.09, p = 0.02) and negatively associated with the predominance of moderate active bouts ($\hat{\beta }$ = -0.21, p = 0.02) in the leisure time composition. Note that the total work time term was not statistically significant in any model (p-values ranging from 0.15 to 0.89). This suggests that the association between work and leisure time movement behaviours was driven by relative and not absolute times.

**Table 4:** Compositional regression analysis estimates: association between leisure time movement behaviour bouts and work time percentage heart rate reserve (%HRR) compositions, stratified by sex.

Variable	$\hat{\beta }$	SE	P-value
**Men (n = 447)**			
ilr_1_(SB ≥30 min)	0.12	0.09	0.19
ilr_1_(SB ≥10–30 min)	0.01	0.05	0.92
ilr_1_(SB <10 min)	0.04	0.05	0.45
ilr_1_(Active<10 min)	0.01	0.04	0.86
ilr_1_(Active≥10 min)	-0.14	0.14	0.32
**Women (n = 356)**			
ilr_1_(SB ≥30 min)	-0.004	0.07	0.95
ilr_1_(SB ≥10–30 min)	0.01	0.07	0.93
ilr_1_(SB <10 min)	0.09*	0.04	0.02
ilr_1_(Active<10 min)	0.05	0.03	0.09
ilr_1_(Active≥10 min)	-0.21*	0.09	0.02

Active = standing, walking, running, stair climbing, and cycling. %HRR = percentage heart rate reserve. SB = sedentary behaviour (sitting and lying).ilr_1_ = first ilr-coordinate, representing the relative importance of a leisure time movement behaviour bout (indicated in parenthesis) with respect to the others. $\hat{\beta }$ = beta-coefficient of the ilr-coordinate of the %HRR composition. Regression models adjusted for age, prescribed heart or lung medicine and average work hours.

*p-value <0.05.

### Compositional isotemporal substitution analyses

Among women, reallocating 15 min to work time spent with ≥40%HRR was associated with an expected increase in short sedentary bouts of 1 min and a decrease in moderate active bouts of 6 min during leisure time (Table 5). Among men, the largest expected change associated with reallocating 15 min to work time spent with ≥40%HRR was found for long sedentary bouts of an increase of 2 min. However, this was not statistically significant (Table 4; p = 0.45).

**Table 5:** Expected change in leisure time movement behaviours bouts associated with reallocating of work time (in minutes) from heart rate reserve (%HRR) below 40% to above 40%, stratified by sex.

Leisure time behaviour	SB*>*30 min			SB *≥*10–30 min			SB<10 min			Active<10 min			Active*≥*10 min		
	Min	95% CI	Δ	Min	95% CI	Δ	Min	95% CI	Δ	Min	95% CI	Δ	Min	95% CI	Δ
**Men (n = 447)**															
Ref. %HRR comp.	96	[35; 219]		106	[47; 213]		64	[30; 116]		72	[36; 153]		87	[20; 221]	
+15 min ≥40%HRR	98	[37; 215]	2	106	[50; 206]	0	64	[32; 115]	0	71	[38; 149]	-1	87	[21; 201]	0
+30 min ≥40%HRR	100	[39; 212]	4	107	[52; 201]	1	65	[33; 112]	1	69	[40; 146]	-3	87	[22; 186]	0
+45 min ≥40%HRR	102	[42; 207]	6	107	[54; 197]	1	65	[34; 112]	1	68	[42; 145]	-4	88	[22; 174]	1
+60 min ≥40%HRR	103	[44; 204]	7	107	[56; 194]	1	66	[36; 111]	2	67	[44; 143]	-5	88	[23; 165]	0
**Women (n = 356)**															
Ref. %HRR comp.	92	[44; 320]		92	[33; 215]		63	[27; 114]		96	[48;146]		144	[39; 234]	
+15 min ≥40%HRR	92	[44; 314]	0	92	[33; 210]	0	64*	[27; 114]	1	97	[49; 145]	1	138*	[41; 232]	-6
+30 min ≥40%HRR	92	[46; 307]	0	92	[34; 204]	0	65*	[28; 112]	2	97	[50; 144]	1	133*	[41; 228]	-11
+45 min ≥40%HRR	91	[47; 305]	-1	91	[35; 202]	-1	66*	[28; 112]	3	98	[51; 144]	2	128*	[41; 224]	-16
+60 min ≥40%HRR	91	[48; 300]	-1	91	[35; 202]	-1	66*	[29; 111]	3	98	[52; 143]	2	125*	[42; 222]	-19

Active = standing, walking, running, stair climbing, and cycling. HRR = heart rate reserve. SB = sedentary behaviour (sitting and lying). Models adjusted for age, prescribed heart or lung medicine and average daily work hours. 95% CI = bootstrap 95% confidence interval for the expected LTPA.

*p-value <0.05. Reference %HRR comp. is the worker’s average %HRR composition.

## Discussion

In this cross-sectional study, we investigated the association between work time spent at high relative aerobic workload, expressed as ≥40%HRR, and movement behaviours during waking leisure time among blue-collar men and women. For an average female worker, reallocating 15 min of work time with <40%HRR to work time with ≥40%HRR was associated with an increase in relative leisure time spent in short sedentary bouts of 1 min and a decrease in moderate active bouts of 6 min. We found no associations among men.

Our finding of a negative association between relative aerobic workload and leisure time activities among women is in line with observations from other cross-sectional studies using device-based measurements [62,63]. One study among 20 female cleaners observed that those with an average relative aerobic workload of ≥25%HRR during work time did not engage in high-intensity physical activities during leisure [62]. Another study assessed the association between workloads at work and leisure time activities derived from METs based on measurements from SenseWear mini armbands among 303 workers (of which 113 were women) [63]. The authors reported that occupational groups with high aerobic workloads at work (mean of 32% VO_2max_) performed the lowest amount of high physical activity at leisure time compared with occupational groups with low- and moderate-aerobic workloads at work (mean of 16% VO_2max_ and 20% VO_2max_, respectively) [63]. Finally, our current findings are in line with our previous study in which the association between occupational and leisure time movement behaviours was investigated using CoDA based on the same study population [54]; here we observed that increasing work time walking by 15 min was associated a decrease in leisure time standing of 7 min among women. Taken together, these findings indicate that women with high aerobic workloads are less likely to have an active leisure time, compared with women with low aerobic workloads.

The expected changes in leisure time of 6 min decreased accumulated time spent in active bouts and 1 min increased accumulated time spent in sedentary bouts among women might seem small. However, replacing 10 min of sedentary time with equal amounts of moderate to vigorous physical activity has been found to lower risk of cardiovascular disease by 12% in a general population [64]. Additionally, replacing 10 min sedentary time with moderate physical activity and vigorous physical activity has shown reduction in risk of metabolic syndrome with 8% and 58%, respectively [65]. Indeed, as little as 1 min replacement of sedentary time with any activity (light, moderate or vigorous) has shown to lower the odds for having metabolic syndrome [65]. Moreover, this group of women were predominantly sedentary during leisure time (Table 2), overweight and with low cardiorespiratory fitness levels (Table 1) and thereby already at increased risk of cardiovascular disease and all-cause mortality [66,67]. Consequently, any decrease in health-enhancing physical activities could have severe health implications for this high-risk population of women.

Among men, we found no association between %HRR at work and leisure time movement behaviours. This result contradicts those of two previous studies. In the first study, the authors observed that high relative aerobic workload of HRR >33% predicted a lower leisure time physical activities during a 4-year follow-up (OR = 0.56, 95% CI = [0.44–0.70]) among 1,891 men from various occupations [26]. Nevertheless, this finding was based on a heterogeneous study population in terms of socioeconomic position and occupation, which could bias the results given that both factors are highly associated with physical job demands and leisure time physical activities [19]. The second study used device-based heart rate measurements over 3–4 days among 42 male construction workers [27]. The authors observed that workers with the highest amount of work time with HRR above 33% had the lowest amount of leisure time spent in high ranges of %HRR, suggesting low levels of moderate to vigorous leisure time physical activities. However, this study did not technically measure physical activities, which limits the comparability with the current results.

### Practical implications

For men and women we observed an average of 42–46 minutes/day of activities ≥40%HRR but low amounts of leisure time physical activities, which is in line with other studies among blue-collar workers [18]. Thus, if not considering if activities are performed at work or leisure, this suggests that the workers are meeting the physical activity guidelines of 150 min of moderate-to-vigorous physical activities per week [21]. However, accumulating evidence indicate that while leisure time physical activities have beneficial health effects, occupational physical activities are likely to have detrimental health effects [1,3,7,9]. Accordingly, interventions addressing health among manual workers should aim at increasing health-enhancing leisure time physical activities and not rely solely on total daily physical activities levels. Unfortunately, such interventions appear to fail in reaching inactive population groups at greatest risk of health impairments [68]. For improving the effect of targeted health interventions, knowledge about determinants for healthy leisure time-use are essential. While several studies have investigated individual determinants for leisure time physical activities [58], little research has been conducted on the effects of physical demands at work on leisure time movement behaviours. However, we argue that a holistic approach considering behaviours both at work and leisure time, like one based on compositional methodology, is required to identify healthy time-use patterns and corresponding determinants among high-risk populations. Furthermore, we suggest that health practitioners and policymakers focus on how work and leisure time activity patterns might impact each other when promoting physical activity guidelines and interventions.

### Strength and limitations

The use of device-based measurements of aerobic workload and leisure time movement behaviours is a strength of this study by limiting misclassification error [28]. The use of exposure variation analyses of leisure time movement behaviours enabled a detailed insight into patterns of active and sedentary bouts. Moreover, the negative association between high aerobic workload at work and active bouts ≥10 min among women supports that exposure variation analyses provided important information beyond that available from data on total time spent in activity categories. Finally, the use of compositional data analysis was a methodological strength which facilitated assessment of the association between relative work time spent with high %HRR and relative leisure time spent on sedentary and active bouts, taking potential interactions between leisure time movement behaviours categories into account. This methodology considerably adds to the field of occupational and public health and physical activity research by enabling research on time-use combinations of physical activities at work and leisure time and health outcomes [69].

The cross-sectional design was a limitation as we cannot rule out an inverse association between low levels of leisure time physical activities and relative aerobic workload. For example, it is plausible that workers performing more leisure time physical activities have higher cardiorespiratory fitness levels and thus, reduced relative aerobic workloads. In this study, we chose not to analyse the pattern of work time spent with uninterrupted periods of high aerobic workload. Consequently, only mean time spent with high %HRR during work time was considered in our analyses. This could be considered a limitation, as different time distribution within %HRR ranges and exertion/rest periods could affect levels of fatigue and thereby leisure time movement behaviours differently [70]. Although the use of EVA enabled detailed insights into leisure time movement patterns, we did not assess the sequences of periods, for instance whether a long, uninterrupted sedentary period was always followed by a short bout of activity. We suggest future studies to potential determinants and health effects of such leisure time movement behaviour sequences. The cut-points for bout-duration and definition set for interrupting a bout were based on current evidence on associations between behaviour bout lengths and health (21–25). Nevertheless, research on this topic is limited and thus the chosen cut-points were not based on solid scientific ground. Finally, the workers in this study had low variation in %HRR during work hours and leisure time physical activities bouts, which could attenuate the strength of the investigated association [71]. Accordingly, the uncertainty of the estimated associations was relatively high given the high standard errors and wide 95% CI.

## Conclusion

In this study we found that a theoretical reallocation of 15 min of work time from <40%HRR to work time spent with ≥40%HRR was associated with a decrease in relative leisure time spent in active bouts of 6 min for the average working women. This finding is of particular concern given that these women were mainly sedentary and consequently, even a few minutes of decrease in active leisure time is likely to impose health impairments. Nevertheless, the result should be interpreted with caution given the relatively high uncertainty in the estimated association. Moreover, our hypothesis of high aerobic workload as a barrier for performing health-enhancing leisure time physical activities was not supported among men.
